# Sodium Ferulate Protects against Angiotensin II-Induced Cardiac Hypertrophy in Mice by Regulating the MAPK/ERK and JNK Pathways

**DOI:** 10.1155/2017/3754942

**Published:** 2017-01-10

**Authors:** Bo Hu, Jian-Tao Song, Xian-Fei Ji, Zun-Qi Liu, Mu-Lin Cong, Dong-Xing Liu

**Affiliations:** Emergency Department, Shandong Provincial Hospital Affiliated to Shandong University, Jinan, Shandong 250021, China

## Abstract

*Background and Objective*. It has been reported that sodium ferulate (SF) has hematopoietic function against anemia and immune regulation, inflammatory reaction inhibition, inhibition of tumor cell proliferation, cardiovascular and cerebrovascular protection, and other functions. Thus, this study aimed to investigate the effects of SF on angiotensin II- (AngII-) induced cardiac hypertrophy in mice through the MAPK/ERK and JNK signaling pathways.* Methods*. Seventy-two male C57BL/6J mice were selected and divided into 6 groups: control group, PBS group, model group (AngII), model + low-dose SF group (AngII + 10 mg/kg SF), model + high-dose SF group (AngII + 40 mg/kg SF), and model + high-dose SF + agonist group (AngII + 40 mg/kg SCU + 10 mg/kg TBHQ). After 7 d/14 d/28 days of treatments, the changes of blood pressure and heart rates of mice were compared. The morphology of myocardial tissue and the apoptosis rate of myocardial cells were observed. The mRNA and protein expressions of atrial natriuretic peptide (ANP), transforming growth factor-*β* (TGF-*β*), collagen III (Col III), and MAPK/ERK and JNK pathway-related proteins were detected after 28 days of treatments.* Results*. SF improved the mice's cardiac abnormality and decreased the apoptosis rate of myocardial cells in a time- and dose-dependent manner (all *P* < 0.05). MAPK/ERK pathway activator inhibited the protective effect of SF in myocardial tissue of mice (*P* < 0.05). SF could inhibit the expression of p-ERK, p-p38MAPK, and p-JNK and regulate the expressions of ANP, TGF-*β*, and Col III (all *P* < 0.05).* Conclusion*. Our findings provide evidence that SF could protect against AngII-induced cardiac hypertrophy in mice by downregulating the MAPK/ERK and JNK pathways.

## 1. Introduction

Cardiac hypertrophy is the common lesion of various cardiovascular diseases, including hypertension, myocardial infarction, and congenital heart disease and also the leading cause of the occurrence of heart failure and sudden death to the patients with the above diseases [1]. The main pathologic changes of cardiac hypertrophy are presented as the changes of many aspects, mainly including myocardial interstitial cell hypertrophy, myocardial interstitial cell proliferation, and extracellular matrix increase of myocardial cells, in other words, myocardial remodeling [2]. Previous studies have found that myocardial apoptosis is simultaneous with myocardial cell hypertrophy and interstitial proliferation and presents the progress of gradual imbalance of apoptosis and proliferation and accelerating heart failure in the process of cardiac hypertrophy (especially cardiac hypertrophy caused by high blood pressure) [3]. The mechanism of cardiac hypertrophy is very complicated. Angiotensin II (AngII) is one of the important factors regulating myocardial cell hypertrophy [4] and its such effect is realized through complex intracellular signal transduction pathways as follows: extracellular signal regulated protein kinase (ERK) is one member of mitogen-activated protein kinase (MAPK) family and also in a close relation with myocardial cell hypertrophy. It is found that the expression level of phosphorylated ERK (p-ERK) commonly existing in the myocardial tissues of patients with pathologic cardiac hypertrophy is increased but its mechanism has not been clearly studied [5, 6].

Sodium ferulate (SF) is the main ingredient of traditional Chinese medicine, Chinese Angelica, and its effects cover hematopoietic function against anemia and immune regulation, inflammatory reaction inhibition, inhibition of tumor cell proliferation, cardiovascular and cerebrovascular protection, and other functions [7]. Studies have reported that SF can regulate MAPK/ERK and JNK pathways [8] and is of application value for AngII-induced cardiac hypertrophy. This research aims to study the protective effect of SF on AngII-induced cardiac hypertrophy of mice and explore its mechanism.

## 2. Materials and Methods

### 2.1. Experimental Animals, Drugs, Primary Instruments, and Reagents

Experimental animals are 72 male C57BL/6J mice at the age of 6–8 weeks supplied by Experimental Animal Center. Drugs are as follows: sodium ferulate (SF) injection solution (Shandong Fangming Pharmaceutical Group, China), AngII (Invitrogen, USA), TBHQ (Selleck, USA), and pentobarbital sodium (Merck, USA). Instruments are as follows: PUMP 11 Elite Injection Pump (Harvard, USA), CODA Noninvasive Blood Pressure Measurement System (Kent scientific Corporation, USA), VEVO2100 Ultrasonic Image System for Small Animals (Visual Sonic, Canada), Microscope (OLYMPUS, Japan), Microplate Reader (Biotek, USA), PCR Instrument (Thermo, USA), Low-Speed Centrifuge (Beckman Coulter, USA), and Gel DoxTM XR + Gel Imaging System (Bio-Rad, USA). Reagents are as follows: RNA extraction and PCR reagents (Roche, Switzerland), TUNEL antibody (Abcam, USA), phosphatase inhibitor (Roche, Switzerland), atrial natriuretic peptide (ANP)/transforming growth factor-*β* (TGF-*β*)/Collagen III (Col III)/bcl-2/bax/caspase-3/ERK/p-ERK/p38MAPK/p-p38MAPK/JNK/p-JNK/*β*-actin antibodies (Cell Signaling Technology, USA), BCA quantitation and Western Blot reagents (Invitrogen, USA), and PCR primers and reagents (Invitrogen, USA). The sequences were as shown in Table 1.

### 2.2. Grouping, Model Building, and Drug Treatments

All mice were cultured for 1 week at room temperature of 20–25°C with circulating air, 12 h illumination, diurnal cycle, and free drinking water and eating. These 30 mice were divided into 6 groups in accordance with random number table, group A is the control group, group B is the injection control group (PBS group), and groups C–F, respectively, refer to the model group (AngII group), model + low-dose SF group (AngII + 10 mg/kg SF group), model + high-dose SF group (AngII + 40 mg/kg SF group), and model + high-dose SF + agonist group (AngII + 40 mg/kg SF + 10 mg/kg tert-butylhydroquinone [TBHQ] group). Weigh all mice after they were fasted for 12 h before operation and then perform mechanical operation after they were given anesthesia and disinfection with intraperitoneal injection of 40 mg/kg pentobarbital sodium. Shear the hair at the location of 2 cm from the mice's middle to posterior back scapula. After disinfecting the shearing location with 75% alcohol, shear longitudinal mouths of 0.5 cm running parallel to the body at the parts close to tails. Use sterilizing forceps to upward isolate the skins and tissues to the location between ears and then downward isolate a little of them. Place the pump cap of micropump containing AngII solution or PBS into the cut towards the mice's heads; after suturing the skin with Stitch 0, put the mice back to cage and out of the operating room after their sobering up. The pumps of mice of groups C–F were perfused with 7.5 mg/ml AngII solution (prepared with PBS containing 0.01 mol/L acetic acid) at the injection dosage of 1.4 mg/kg/d for 28 d while the pumps of mice of group B were perfused with PBS solution, and the injection dosage and speed were adjusted to the same with groups C–F. Perform no operation for group A. Give daily subcutaneous injection of 10 mg/kg SF to the mice of group D (prepared with physiological saline water), 40 mg/kg SF to group E, and 40 mg/kg SF and 10 mg ERK activator TBHQ to group F and simultaneously give equivalent physiological saline water to the mice of group B and group C.

### 2.3. Detection of Blood Pressure, Heart Rate, and Ultrasonic Cardiogram of the Mice

Basic blood pressure and heart rates of mice were measured before operation. Then use Noninvasive Blood Pressure Measurement Analysis System to, respectively, measure the mice's systolic blood pressure (SBP), diastolic blood pressure (DBP), and heart rate (HR) at 7 d, 14 d, and 28 d after operation and record the changes of the above indexes. Use Doppler Color Ultrasonic Diagnosis Instrument to examine the mice's ultrasonic cardiogram as follows: Use deshedding cream to shed off the hair at the location inclined left to the junction of chest and abdomen after anesthesia; then, respectively, make horizontal measurements of posterior wall thickness at diastole (PWTD), posterior wall thickness at diastole telophase (PWTS), left ventricular end diameter at diastole telophase (LVEDD), and interventricular septal thickness at diastole (IVSTD) in mitral valve cavity under the circumstance of free breathing and then take the average value after measuring each index for 3 consecutive cardiac cycles.

### 2.4. Observation of Cardiac Morphology and Detection of Apoptosis Rate of Myocardial Cells of the Mice

Kill the mice and fix them in supine position. Then cut open the location along the xiphoid to the armpits under two sides and take out their hearts. Put them into cold PBS liquid to rinse off residual blood and suck them dry with filter paper. Next, weigh the hearts with ten-thousandth balance scale and calculate the ratio of heart and body weight. The ratio of heart and body weight = the mice's heart weight (HW)/body weight (BW) × 100.00%. Cut off part of the left ventricles of hearts and fix them with 4% paraformaldehyde. Then take out the tissues from the fixed liquid and conduct gradient alcohol dehydration in accordance with the following sequences: 70% alcohol (1 h), 80% (1 h), 95% alcohol (twice, 1 h/time), and 100% alcohol (three times, 0.5 h/time, 0.5 h/time, and 1 h/time) (dimethylbenzene infusion (twice, 1 h/time); 65°C paraffin infusion for 3-4 h). Slice them after inclusion. Then take paraffin slices at the size of 3-4 *μ*m and mount them onto glass slide for back-up supply. Dewax paraffin slices with routine dimethylbenzene and rinse them with gradient alcohol to hydration. Dip-dye them with hematoxylin for 15–30 min and then rinse them with water. Differentiate them with 1% hydrochloric acid alcohol and then rinse them with water. Blue them with saturated lithium carbonate and then rinse them under running water for 15–30 min. Dip-dye them with 1% soluble eosin for 5–15 s. Conduct routine dehydration, transparency, and sealing sheet, and then observe the morphological changes of heart tissues under microscope. Measure the surface area of cells with the control group as 1 and the areas of cells of other groups were equivalent to the measuring of areas of myocardial cells/areas of myocardial cells of the control group. Dry them for 1 h at 60°C with paraffin slices and then hydrate them. Place the slices into 0.01 mol/L citric acid buffer for 10 min microwave heating to repair antigen and then incubate them with 0.3% Triton-X100 for 30 min. Rinse them under running water for 5 min and then with PBS at 5 min each time for 3 times. Wipe dry the extra moisture around tissues and then dropwise add 50 *μ*l TUNEL reaction mixture (dropwise add in the mixture of 50 *μ*l TdT and 450 *μ*l fluorescein-labelled dUT for the treatment group; only 50 *μ*l fluorescein-labelled dUT liquid for negative control group; and first dropwise add in 100 *μ*l DNase I for reaction at 25°C for 10 min and then the same procedures with the treatment group for positive control group). Place the slices into a dark moist chamber at 37°C for 1 h and then bleach and rinse them with PBS at 5 min each time for 3 times. Incubate the slices with phalloid (at 1 : 50 concentration) for 1 h under room temperature. After bleaching and rinsing them with PBS at 5 min each time for 3 times, conduct sealing sheet with anticancellation reagent containing DAPI. The cells with brownish black granules observed under microscope were apoptotic myocardial cells. Randomly select 5 visions (200 times) from the slices of each group. Calculate the amount of apoptotic cell nucleuses and myocardial cells as well as the apoptosis index (apoptosis rate = apoptotic cells/the total number of myocardial cells) of myocardial cells and then take their average value. Repeat this experiment for three times.

### 2.5. Detection of Expression Levels of Myocardial Tissues (Bcl-2, Bax, and Caspase-3) of the Mice

Take 30 mg myocardial tissues and cut them into pieces with scissors. Then place them into 2 ml EP tube and extract the total RNA in accordance with the specification of TRIzol™ kit. Determine the concentration and purity of RNA and then take 1 *μ*g of the total RNA and reverse transcript them into cDNA. Next, take 5 *μ*l cDNAxo and dilute it into it 4 times. Take 2 *μ*l as the template and, respectively, conduct Q-PCR with the primers of target genes and *β*-actin GAPDH under such reaction conditions: perform denaturation for 3 s at 95°C; perform denaturation for 10 s at 95°C; perform annealing/extend for 30 s at 60°C, and repeat this for 40 cycles; read the plate for 5 S at each increase of 0.5°C ranging from 65 to 95°C to form the dissolution curve. Set up 3 parallel tubes for all samples and express the quantity of gene expression with the average cycle-threshold value (Ct) of parallel tube. Calculate relative expression quantity of bcl-2, bax, and caspase-3 by the application of 2^−ΔΔCt^. Take 30 mg myocardial tissues and cut them into pieces with scissors. Then place them into 2 ml EP tube. Add in 60 *μ*l 2% SDS Protein Lysis Buffer (preadd in phosphatase inhibitor) and then conduct mechanical homogenate till there is no residual tissue block (on-ice operation). Centrifuge them at 14000 spins/min for 10 min (at 4°C) and then place them under −80°C for conservation after protein subpackage. After measuring the concentration by BCA method, respectively, take 30 *μ*g protein to perform polyacrylamide gel electrophoresis and 60~90 min 100 V constant-pressure transmembrane; Seal them at room temperature for 1~2 h with 5% nonfat dried milk/TBST and then add in 1 : 1000 bcl-2/bax/caspase-3 antibody for overnight incubation at 4°C. Perform 1 : 5000 *β*-actin incubation at room temperature for 1.5 h and TBST membrane cleaning at 10 min × 3 times. Then incubate them at room temperature for 2 h with horse radish peroxidase labelled goat-anti-rabbit/anti-mouse second antibody. Next, conduct TBST membrane cleaning at 10 min × 2 times and TBS membrane cleaning for 5 min. ECL presented the color of luminous kit and image development and formation by the application of DOXTM XR + Gel Imaging System. Take the ratio of each band's euphotic volume [9] and *β*-actin band as the relative expression quantity of target protein. Repeat this experiment for three times.

### 2.6. Detection of Expression Levels of Myocardial Tissues (ANP, TGF-*β*, and Col III) of the Mice

Apply Q-PCR and Western Blot to detect the expression levels of mRNA and protein of myocardial tissues including ANP (antibody 1 : 1000), TGF-*β* (antibody 1 : 1000), and Col III (antibody 1 : 1000) in accordance with the procedures specified in 1.5. Repeat this experiment for three times.

### 2.7. Detection of Expression Levels of Myocardial Tissues (ERK, p-ERK, and p38MAPK) of the Mice

Apply Western Blot to detect the protein expression level of myocardial tissues including ERK (antibody 1 : 3000), p-ERK (antibody 1 : 1000), p38MAPK (antibody 1 : 2000), p-p38MAPK (antibody 1 : 1000), JNK (antibody 1 : 3000), and p-JNK (antibody 1 : 1000) in accordance with the procedures specified in 1.5. Repeat this experiment for three times.

### 2.8. Statistical Analysis

Use SPSS19.0 Statistical Software to make single-factor variance analysis of measurement data, and all analytical data results are expressed as mean ± standard deviation ($\overset{-}{x}\pm SD$). The data with its difference *P* < 0.05 is regarded to be of statistical significance.

## 3. Results

### 3.1. Changes of Blood Pressure and Heart Rate of the Mice in Each Group

After 28 days of treatment, the heart rates and SBP of the mice in the AngII group, AngII + 10 mg/kg SF group, and AngII + 40 mg/kg SF + 10 mg/kg TBHQ group were significantly increased compared with before treatment (*P* < 0.05) and remarkably higher than the control group and PBS group (*P* < 0.05). Compared with the control group and PBS group, AngII + 40 mg/kg SF group showed no significant change (*P* < 0.05). Compared with AngII group, the heart rates and SBP of AngII + 10 mg/kg SF group and AngII + 40 mg/kg SF group were significantly decreased (*P* < 0.05). DBP differences of the mice of each group at different moments indicated no statistical significance (*P* > 0.05). See Table 2.

### 3.2. Detection Results of HW/BW and Ultrasonic Cardiogram of the Mice in Each Group

Compared with the control group, HW/BW, PWTS, LVEDD, and IVSTD of PBS group showed no significant change (*P* > 0.05). Compared with the control group and PBS group, the above indexes of AngII group and AngII + 40 mg/kg SF + 10 mg/kg TBHQ group were remarkably increased (*P* < 0.05) while the above indexes of AngII + 40 mg/kg SF group were significantly lower than AngII group (*P* < 0.05). PWTS differences of the mice of each group indicated no statistical significance (*P* > 0.05). See Table 3.

### 3.3. Surface Areas of Myocardial Cells of the Mice in Each Group

**Figure 1:**
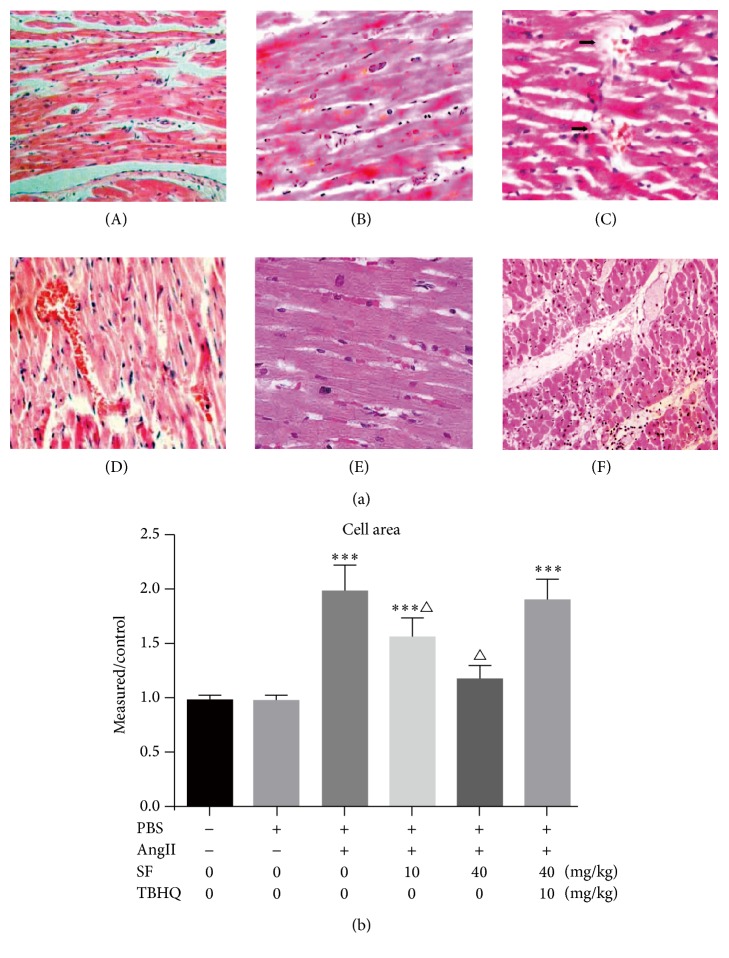
The myocardial tissue sections of different groups (HE stain, ×400). (a) HE staining showed the morphologies of different groups. ((a)-(A)) Control group; ((a)-(B)) PBS group; ((a)-(C)) AngII group; ((a)-(D)) AngII + 10 mg/kg group; ((a)-(E)) AngII + 40 mg/kg SF group; ((a)-(F)) AngII + 40 mg/kg SF + 10 mg/kg TBHQ group. (b) The cell area of different groups. Scale bar = 50 *μ*m, ^*∗∗∗*^*P* < 0.01 versus control, ^△^*P* < 0.05 versus Ang II, and *n* = 3.

The arrangements of myocardial cells of the mice of the control group (Figure 1(a)-(A)), PBS group (Figure 1(a)-(B)), and AngII + 40 mg/kg SF group (Figure 1(a)-(E)) were in order, cell nucleuses presented blue staining, and cytoplasm presented red staining. Compared with the above three groups, the mice of AngII group (Figure 1(a)-(C)) and AngII + 40 mg/kg SF + 10 mg/kg TBHQ group (Figure 1(a)-(F)) presented larger-grown myocardial cells, loose cell arrangements, and increased intercellular matrixes; AngII + 10 mg/kg SF group showed increased cell mildness while its intercellular matrix presented no significant abnormality (Figure 1(a)-(D)). Compared with the control group, the cell surface area of PBS group indicated no significant change (*P* > 0.05). Compared with the control group and PBS group, the above indexes of AngII group and AngII + 40 mg/kg SF + 10 mg/kg TBHQ group were notably increased (*P* < 0.05) and the above indexes of AngII + 40 mg/kg SF group were significantly lower than AngII group (*P* < 0.05). See Figure 1(b).

### 3.4. Detection of Myocardial Cell Apoptosis of the Mice in Each Group

**Figure 2:**
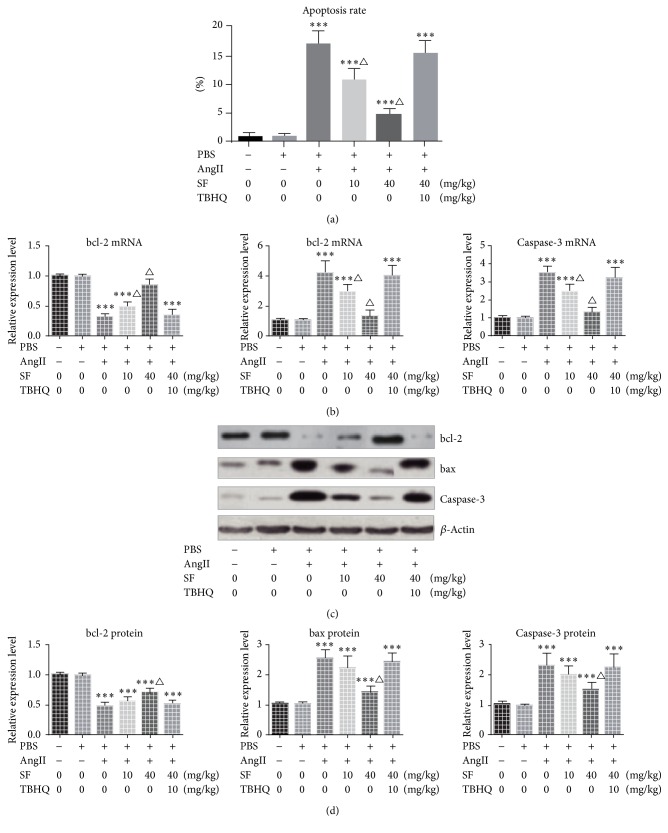
The apoptosis analysis of different groups. (a) The apoptosis rate analysis and (b) the comparison of mRNA expression. ((c) and (d)) Western Blot showed the expression level of apoptosis related proteins. ^*∗∗∗*^*P* < 0.01 versus control, ^△^*P* < 0.05 versus Ang II, and *n* = 3.

Compared with the control group, the cell apoptosis rate of PBS group presented no significant change (*P* > 0.05). The cell apoptosis rate and expression levels of bax and caspase-3 of the mice of groups injected AngII were notably increased (*P* < 0.05) while the protein expression level of bcl-2 was remarkably decreased (*P* < 0.05). Compared with the control group, the mRNA levels of bcl-2, bax, and caspase-3 of the mice of AngII + 40 mg/kg SF group showed no significant change (*P* > 0.05). Compared with AngII group, the cell apoptosis rates and mRNA expression levels of bax and caspase-3 of AngII + 10 mg/kg SF group and AngII + 40 mg/kg SF group were remarkably decreased (*P* < 0.05) while the mRNA level of bcl-2 was notably increased (*P* < 0.05). Compared with AngII group, the cell apoptosis rate and mRNA and protein levels of bcl-2, bax, and caspase-3 of AngII + 40 mg/kg SF + 10 mg/kg TBHQ group presented no significant change (*P* > 0.05). See Figure 2.

### 3.5. Expression Levels of Myocardial Tissues (ANP, TGF-*β*, and Col III) of the Mice in Each Group

**Figure 3:**
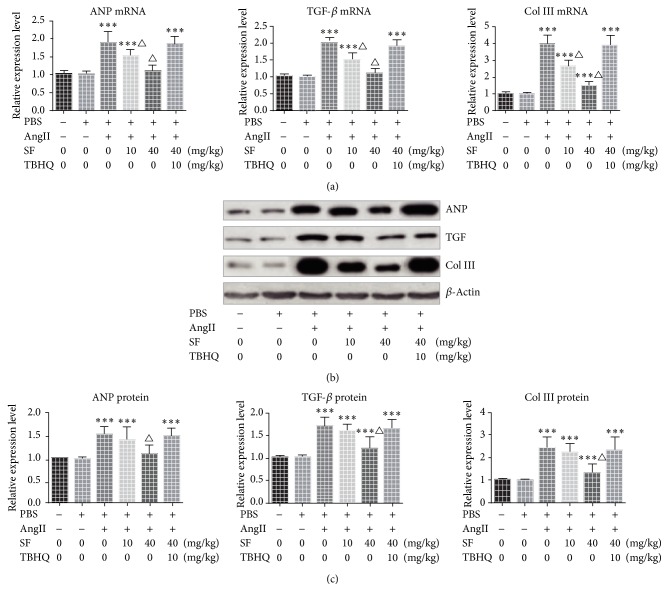
The mRNA expressions of ANP, TGF-*β*, and Col III in each group. (a) Q-PCR detected the expression of mRNA; ((b) and (c)) Western Blot showed the expression level of proteins. ^*∗∗∗*^*P* < 0.01 versus control, ^△^*P* < 0.05 versus Ang II, and *n* = 3.

Compared with the control group, the expression levels of ANP, TGF-*β*, and Col III of the mice of PBS group presented no significant change (*P* > 0.05) while the expression levels of the above genes of AngII group, AngII + 10 mg/kg SF group, and AngII + 40 mg/kg SF + 10 mg/kg TBHQ group were notably increased (*P* > 0.05). The expression level of ANP of AngII + 40 mg/kg SF group presented no significant change (*P* > 0.05) while the expression levels of TGF-*β* and Col III were significantly increased compared with the control group (*P* < 0.05). Compared with AngII group, mRNA levels of ANP, TGF-*β*, and Col III of AngII + 10 mg/kg SF group were notably decreased (*P* < 0.05) while its protein level showed no significant change (*P* > 0.05). The mRNA and protein levels of the above genes of AngII + 40 mg/kg SF group were significantly decreased (*P* < 0.05). See Figure 3.

### 3.6. Activation of MAPK/ERK and JNK Pathways of Myocardial Tissues of the Mice in Each Group

**Figure 4:**
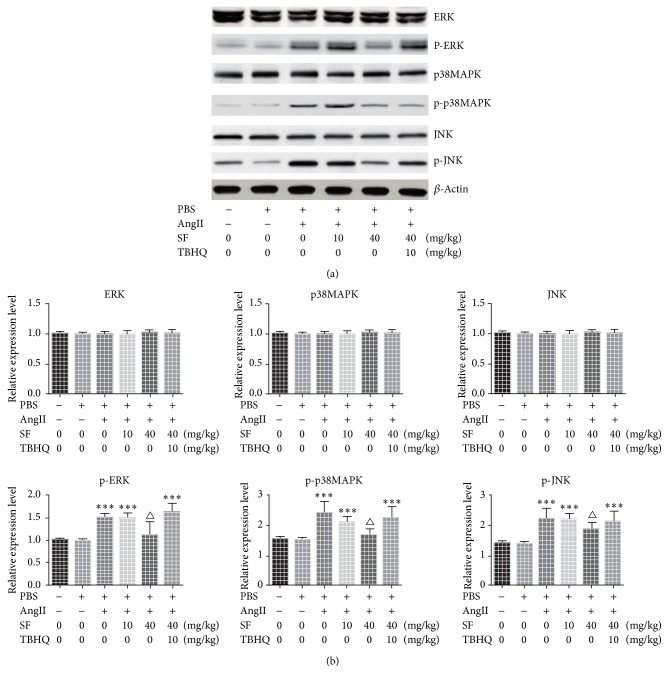
The expressions of ERK, p-ERK, p38MAPK, p-p38MAPK, JNK, and p-JNK in each group. ^*∗∗∗*^*P* < 0.01 versus control, ^△^*P* < 0.05 versus Ang II, and *n* = 3.

The expressions of ERK/p38MAPK/JNK of mice in each group presented no significant change after treatment (all *P* > 0.05). Compared with the control group, the expressions of p-ERK, p-p38MAPK, and p-JNK of the mice of PBS group and Ang II + 40 mg/kg SF group showed no significant change (*P* > 0.05) and were notably lower compared to AngII group (*P* < 0.05). The expressions of p-ERK, p-p38MAPK, and p-JNK of mice of AngII group, AngII + 10 mg/kg SF group, and AngII + 40 mg/kg SF + 10 mg/kg TBHQ group were significantly increased (*P* < 0.05) compared with the control group. See Figure 4.

## 4. Discussion

AngII is the most important active ingredient in renin-angiotensin system and also an important humoral factor to regulate cardiac hypertrophy. Studies on the levels of whole body and organs as well as experiments on cultured cells have successively confirmed that AngII has a direct effect on promoting cardiac hypertrophy [10, 11]. This study used the method of continuous-pumping-AngII into the backs of male C57BL/6 mice to establish the model of cardiac hypertrophy and the control group without placing-in micropumps. The heart pressure and heart rate of the mice of AngII group were significantly increased after 14–28 d, and their heart and weight indexes, PWTS, LVEDD, and IVSTD, were notably higher compared to the control group, which showed that the model of cardiac hypertrophy caused by AngII in this study was successfully established.

The typical pathological change of cardiac hypertrophy is myocardial remodeling, including myocardial cell hypertrophy, myocardial interstitial cell proliferation, and extracellular matrix increasing [12]. In this study, myocardial cell area growing, intercellular matrix increasing, Col III expression level rising, and other typical pathological changes were all found in the AngII treated mice; we also found the apoptosis rate of myocardial cells increased, the expression level of bax and caspase-3 increased (myocardial tissues), and the expression level of bcl-2 decreased compared with the control group. Previous studies have found that the apoptosis of myocardial cells plays certain role in the process of pathological cardiac hypertrophy probably because of the existence of negative feedback regulation in the process of tissue proliferation and apoptosis while the increase of levels of AngII and other tissue factors can stimulate the synthesis of proteins related to activating the apoptosis genes leading to promoting the apoptosis of myocardial cells and further decreasing myocardial contraction force and deficient pump blood [13, 14]. Myocardial cells are terminally differentiated somatic cells; therefore, they cannot meet the needs of repair damaged heart relying on the proliferation of these cells. Instead, organism starts a series of adjustments to maintain heart structure by stimulating protein synthesis of myocardial cells, fibroblast proliferation, and collagen increase, namely, the pathological process of cardiac hypertrophy [15, 16].

As one member of MAPK signal transduction enzyme superfamily, ERK is an important signaling molecule in regulating cell growth, proliferation, and differentiation [17, 18]. In myocardial cells, AngII can activate p38MAPK pathway and the functions of 2 isomers of p38 are interconstraint. The activation of p38-*β*MAPK can induce the hypertrophy of myocardial cells while the activation of p38-*α*MAPK can antagonistically rival against the above function and lead to cell apoptosis [19]. In this study, the level of p-ERK and p-p38MAPK of the mice in AngII group was increased. However, this study did not further explore the competition results of these two subtypes. Therefore, there was still no conclusion for the final effect of p38MAPK.

Treating the mice (they were given continuous pumping-in AngII) with SF at different doses, we have found the following: SF controls the rise of blood pressure and heart rate in a time- and dose-dependent manner compared with the mice in AngII group. HW/BW, PWTS, IVSTD, myocardial cell area, cell apoptosis level, and Col III of AngII + 10 mg/kg SF group and AngII + 40 mg/kg SF group were remarkably lower compared to the mice in AngII group; however, the above indexes of AngII + 40 mg/kg SF + 10 mg/kg TBHQ group presented no significant change compared with the AngII group. These data indicated that SF can control the cardiac hypertrophy caused by AngII in a time- and dose-dependent manner and this effect can be blocked by ERK agonists which demonstrated that SF can inhibit cardiac myocyte hypertrophy by regulating MAPK/ERK and JNK pathways. Previous studies have found that SF can inhibit the apoptosis of ischemic and hypoxic neurons by activating MAPK/ERK and JNK pathways [20]. In this study, it is found that SF can inhibit ERK possibly because the MAPK/ERK and JNK pathways in AngII-induced cardiac hypertrophy and ischemic and hypoxic damage have pathological difference while SF can protect damaged cells by regulating ERK.

The study has also found that the expression levels of atrial natriuretic peptide (ANP) and TGF-*β* of the mice in AngII group are significantly increased compared with the control group and PBS group. The rise of AngII is followed by compensated rise of ANP in a positive correlation with the level of AngII without the ability to maintain the balance of vascular active substances [21]. TGF-*β* is in close relation with the occurrence of cardiac hypertrophy. In vitro studies have shown that TGF-*β* can promote the generation of extracellular matrix and the differentiation of fibroblasts [22]. In this study, we found that SF can inhibit the expression of TGF-*β* as well as suppressing ERK activation indicating that these effects may be interactive and more studies needed.

In conclusion, SF can inhibit cardiac hypertrophy caused by AngII and its effect may be associated with MAPK/ERK and JNK pathways.

## Figures and Tables

**Table 1:** The sequence of PCR primer of detected genes.

Genes	Primer	Sequence (5′-3′)
ANP	Forward	GTGCGGTGTCCAACACAGAT
	Reverse	TCCAATCCTGTCAATCCTACCC
TGF-*β*	Forward	CTTCGACGTGACAGACGCT
	Reverse	GCAGGGGCAGTGTAAACTTATT
Col III	Forward	GTGGCTTTGGTCCTATCTGTC
	Reverse	CGTGTGTGAAATGTCATTGATCC
bax	Forward	AGACAGGGGCCTTTTTGCTAC
	Reverse	AATTCGCCGGAGACACTCG
bcl-2	Forward	GCTACCGTCGTGACTTCGC
	Reverse	CCCCACCGAACTCAAAGAAGG
Caspase-3	Forward	CTCGCTCTGGTACGGATGTG

**Table 2:** The comparison of HR, SBP, and DBP among different groups ($\overset{-}{x}\pm SD$).

Indexes	Times (d)	Control	PBS	Ang II	Ang II + 10 mg/kg SF	Ang II + 40 mg/kg SF	Ang II + 40 mg/kg SF + 10 mg/kg TBHQ
HR (beats/times)	0	471.9 ± 24.5	477.3 ± 25.4	473.8 ± 25.6	469.9 ± 27.4	471.8 ± 22.6	474.5 ± 26.8
	7	472.4 ± 25.8	475.8 ± 26.3	488.2 ± 31.2	479.6 ± 23.5	477.9 ± 24.8	486.5 ± 26.4
	14	476.2 ± 29.8	474.6 ± 27.8	507.7 ± 25.5^*∗∗∗*#^	488.5 ± 27.6	481.2 ± 22.9^Δ^	508.4 ± 18.6^*∗∗∗*#^
	28	476.9 ± 27.4	481.9 ± 31.6	523.9 ± 27.8^*∗∗∗*#^	501.9 ± 23.8^*∗∗∗*Δ#^	479.9 ± 23.5^Δ^	519.9 ± 19.6^*∗∗∗*#^

SBP (mmHg)	0	115.4 ± 9.8	116.4 ± 9.5	115.2 ± 10.1	117.4 ± 9.5	115.9 ± 8.6	116.9 ± 11.4
	7	116.6 ± 10.5	117.8 ± 11.2	121.8 ± 11.4	118.9 ± 10.5	117.4 ± 9.7	122.0 ± 12.2
	14	118.2 ± 11.6	117.5 ± 10.5	127.5 ± 10.3^*∗∗∗*#^	122.4 ± 11.3	119.9 ± 10.8^Δ^	126.8 ± 9.8^*∗∗∗*#^
	28	117.8 ± 11.2	119.7 ± 10.6	139.5 ± 10.6^*∗∗∗*#^	127.8 ± 9.9^*∗∗∗*Δ#^	121.9 ± 11.6^Δ^	137.5 ± 12.6^*∗∗∗*#^

DBP (mmHg)	0	82.8 ± 8.1	81.6 ± 7.8	83.9 ± 8.0	84.1 ± 8.5	84.4 ± 8.2	85.2 ± 9.4
	7	84.5 ± 8.2	82.5 ± 7.9	85.5 ± 9.1	84.9 ± 8.4	85.1 ± 8.9	86.5 ± 10.1
	14	83.1 ± 8.5	84.0 ± 8.4	86.9 ± 9.6	85.2 ± 9.2	85.5 ± 9.2	85.9 ± 9.5
	28	84.1 ± 7.9	83.3 ± 8.1	88.2 ± 11.4	87.1 ± 10.2	86.2 ± 9.8	84.9 ± 9.2

*Note.* Compared with Sham group, the difference had statistical significance, ^*∗∗∗*^*P* < 0.01.

Compared with PBS group, the difference had statistical significance, ^*∗∗∗*^*P* < 0.01.

Compared with Ang II group, the difference had statistical significance, ^Δ^*P* < 0.05.

Compared with that at 0 d, the difference had statistical significance, ^#^*P* < 0.05.

**Table 3:** The compare of HW/BW and UCG results among different groups ($\overset{-}{x}\pm SD$).

Indexes	Control	PBS	Ang II	Ang II + 10 mg/kg SF	Ang II + 40 mg/kg SF	Ang II + 40 mg/kg SF + 10 mg/kg TBHQ
HW/BW (mg/g)	6.17 ± 0.09	6.24 ± 0.11	7.08 ± 0.14^*∗∗∗*^	6.69 ± 0.13^*∗∗∗*^	6.47 ± 0.08^*∗∗∗*Δ^	7.04 ± 0.18^*∗∗∗*^
PWTD (mm)	0.81 ± 0.05	0.79 ± 0.06	0.97 ± 0.10^*∗∗∗*^	0.90 ± 0.07^*∗∗∗*Δ^	0.82 ± 0.05^Δ^	0.95 ± 0.12^*∗∗∗*^
PWTS (mm)	1.24 ± 0.06	1.25 ± 0.07	1.29 ± 0.06	1.27 ± 0.04	1.25 ± 0.05	1.28 ± 0.08
LVEDD (mm)	3.12 ± 0.09	3.15 ± 0.10	2.98 ± 0.08^*∗∗∗*^	3.00 ± 0.06^*∗∗∗*^	3.09 ± 0.11^Δ^	2.98 ± 0.10^*∗∗∗*^
IVSTD (mm)	1.42 ± 0.06	1.43 ± 0.05	1.52 ± 0.05^*∗∗∗*^	1.47 ± 0.04^*∗∗∗*Δ^	1.43 ± 0.06^Δ^	1.50 ± 0.05^*∗∗∗*^

*Note.* Compared with Sham group, the difference had statistical significance, ^*∗∗∗*^*P* < 0.01.

Compared with PBS group, the difference had statistical significance, ^*∗∗∗*^*P* < 0.01.

Compared with Ang II group, the difference had statistical significance, ^Δ^*P* < 0.0.
